# Validity and repeatability of cardiopulmonary exercise testing in interstitial lung disease

**DOI:** 10.1186/s12890-022-02289-0

**Published:** 2022-12-22

**Authors:** Owen W. Tomlinson, Laura Markham, Rebecca L. Wollerton, Bridget A. Knight, Anna Duckworth, Michael A. Gibbons, Chris J. Scotton, Craig A. Williams

**Affiliations:** 1 Department of Public Health and Sports Sciences, Faculty of Health and Life Sciences, University of Exeter, Heavitree Road, Exeter, EX1 2LU UK; 2 Academic Department of Respiratory Medicine, Royal Devon University Healthcare NHS Foundation Trust, Barrack Road, Exeter, EX2 5DW UK; 3 Department of Clinical and Biomedical Science, Faculty of Health and Life Sciences, University of Exeter, Heavitree Road, Exeter, EX1 2LU UK; 4 NIHR Exeter Clinical Research Facility, Royal Devon University Healthcare NHS Foundation Trust, Barrack Road, Exeter, EX2 5DW UK

**Keywords:** Aerobic fitness, Clinical physiology, Maximal exercise, Pulmonary disease

## Abstract

**Background:**

Cardiopulmonary exercise testing (CPET), and its primary outcome of peak oxygen uptake (VO_2peak_), are acknowledged as biomarkers in the diagnostic and prognostic management of interstitial lung disease (ILD). However, the validity and repeatability of CPET in those with ILD has yet to be fully characterised, and this study fills this evidence gap.

**Methods:**

Twenty-six people with ILD were recruited, and 21 successfully completed three CPETs. Of these, 17 completed two valid CPETs within a 3-month window, and 11 completed two valid CPETs within a 6-month window. Technical standards from the European Respiratory Society established validity, and repeatability was determined using mean change, intraclass correlation coefficient and typical error.

**Results:**

Every participant (100%) who successfully exercised to volitional exhaustion produced a maximal, and therefore valid, CPET. Approximately 20% of participants presented with a plateau in VO_2_, the primary criteria for establishing a maximal effort. The majority of participants otherwise presented with secondary criteria of respiratory exchange ratios in excess of 1.05, and maximal heart rates in excess of their predicted values. Repeatability analyses identified that the typical error (expressed as percent of coefficient of variation) was 20% over 3-months in those reaching volitional exhaustion.

**Conclusion:**

This work has, for the first time, fully characterised how patients with ILD respond to CPET in terms of primary and secondary verification criteria, and generated novel repeatability data that will prove useful in the assessment of disease progression, and future evaluation of therapeutic regimens where VO_2peak_ is used as an outcome measure.

## Introduction

Interstitial lung disease (ILD) is the collective term for a series of pulmonary disorders characterised by inflammation, interstitial and alveolar damage, and irreversible declines in lung function [1]. Presently, ILD affects approximately 2 million people [2] and results in approximately 120,000 deaths globally [3]. Traditionally, resting measures of pulmonary function, including forced vital capacity (FVC) and the diffusion capacity of carbon monoxide (DL_CO_), have been utilised to monitor disease progression and evaluate the efficacy of treatments. Both variables are predictive of mortality [4] and provide greater predictive power for survival over 6 months than histopathological factors alone [5]. However, these are not the sole factors predictive of mortality.

Cardiopulmonary exercise testing (CPET) is a dynamic diagnostic and prognostic test that simultaneously stresses multiple organ systems in order to identify causes of exercise intolerance, and obtain functionally useful biomarkers [6]. Lower values for peak oxygen uptake (VO_2peak_), the primary outcome from cardiopulmonary exercise testing (CPET), are also associated with increased risk of mortality and need for transplantation [7–10], enhancing the predictive power of static pulmonary function testing [8], whilst also maintaining high independent predictive power when these factors are controlled for [7]. Ventilatory efficiency, and exercise induced hypoxemia are also indicative of poorer prognosis [11], thus highlighting the importance of more functionally derived data available from CPET as independent and dynamic prognostic outcome measures in addition to traditional, static, pulmonary variables.

The utility and validity of CPET in a range of pulmonary disease has been described previously [12], and within the key requirements of exercise protocols eliciting VO_2peak_ is confidence that a ‘maximal’ value has been achieved and that sub-maximal values are not mistakenly accepted [12]. However, of previous studies to utilise CPET in ILD, it is unclear as to whether maximal exercise has actually been achieved, as prior studies in ILD have either not reported how maximal exercise is classified [7–9], or only use limited criteria to establish a ‘maximal’ value [10]. Equally, there is a lack of data on the repeatability of CPET in ILD, with a need to understand this to be able to accurately interpret significant and clinically meaningful changes in function, to inform and evaluate treatment options and appropriately assess disease progression [13].

Therefore, this study sought to characterise CPET responses in patients with ILD, focusing on the validity and repeatability of the test, with particular emphasis with regards to VO_2peak_, to further the evidence for the using this parameter as an independent physiological marker of disease progression in ILD.

## Materials and methods

### Study design, population and ethics

This analysis forms part of a wider study (PETFIB: *Exploring the potential of Cardio-****P****ulmonary ****E****xercise ****T****esting as a biomarker in patients diagnosed with ****FIB****rosing Lung Disease*), whereby the clinical feasibility and patient acceptability of CPET, and initial results on participant characteristics, have been previously reported [14]. This study recruited 26 people with ILD [19 male] via convenience sampling, of differing diagnoses, and prescribed differing medications as per Table 1. All participants attended the research facility on three occasions, over a 20-month period from August 2017 to May 2019, with a period of 3 months (0.2–0.3 years) separating each visit where possible.

**Table 1:** Baseline anthropometric, pulmonary, and clinical data in study participants

Variable	All (*n* = 26)	Min 1 × CPET (*n* = 24)	3 × CPET (*n* = 21)
Male/Female (n)	19/7	17/7	17/4
Age (years)	70.3 ± 7.7	69.7 ± 7.6	69.8 ± 7.6
Stature (cm)	171 ± 7	170 ± 7	171 ± 7
Mass (kg)	80.5 ± 13.9	81.0 ± 14.4	82.5 ± 13.4
BMI (kg^.^m^−2^)	27.6 ± 3.8	27.8 ± 3.8	28.1 ± 3.6
Body Fat (%)	36.8 ± 10.1	37.1 ± 10.4	35.8 ± 10.3
Fat Mass (kg)	29.9 ± 10.1	30.3 ± 10.4	30.0 ± 10.6
Fat Free Mass (kg)	50.6 ± 10.1	50.7 ± 10.4	52.5 ± 9.1
FEV_1_ (L)	2.40 ± 0.54^b^	2.39 ± 0.54^b^	2.48 ± 0.49^b^
FEV_1_ (%_Pred_)	86.4 ± 14.6^b^	86.3 ± 14.9^b^	86.9 ± 15.2^b^
FVC (L)	3.06 ± 0.77^b^	3.04 ± 0.78^b^	3.18 ± 0.70^b^
FVC (%_Pred_)	84.2 ± 16.7	84.0 ± 17.3	84.4 ± 17.8
FEV_1_/FVC	0.79 ± 0.08^b^	0.80 ± 0.07^b^	0.79 ± 0.09^b^
DL_CO_ (mL min^−1^ kPa^−1^)	4.48 ± 1.09^c^	4.54 ± 0.93^c^	4.63 ± 0.99^d^
DL_CO_ (%_Pred_)	54.8 ± 12.9^a^	55.4 ± 11.6^a^	55.2 ± 12.3^a^
GAP Score	4 ± 1	4 ± 1	4 ± 1
Physical activity level^*^	2 ± 1	2 ± 1	2 ± 1
*Antifibrotic use*			
Nintedanib	10	9	8
Pirfenidone	3	3	3
*Diagnosis*			
IPF	19	17	15
CHP	3	3	2
UIP	2	2	2
Probable IPF	1	1	1
Organising pneumonia	1	1	1

All continuous variables reported as mean ± standard deviation. Categorical data presented as whole numbers

*Physical Activity scaled from 1 to 4 (1, inactive; 2, moderately inactive; 3, moderately active; 4; active)

*BMI* body mass index; *IPF* idiopathic pulmonary fibrosis; *UIP* usual interstitial pneumonia; *CHP* chronic hypersensitivity pneumonitis; *FEV*_*1*_ forced expiratory volume in one second; *FVC* forced vital capacity; *DL*_*CO*_ diffusion capacity for carbon monoxide; *GAP* gender-age-physiology score; *CPET* cardiopulmonary exercise test

^a^ = *n*–1

^b^ = *n*–2

^c^ = *n*–4

^d^ = *n*–6

Ethics approval for this study was granted by the Health Research Authority (IRAS 220189) following review by the South West (Frenchay) Research Ethics Committee (17/SW/0059). All participants provided written and informed consent upon recruitment to the study.

### Physiological measures

Participant’s stature and body mass were assessed using standard methods, with body mass index (BMI) subsequently calculated. Body fat percentage was assessed using air displacement plethysmography (BodPod; COSMED, Rome, Italy), with subsequent values for fat mass and fat-free mass (FFM) calculated.

Retrospective measures of pulmonary function were obtained from medical records, whereby the date closest to the participants first CPET was utilised. Measures included forced expiratory volume in one second (FEV_1_), FVC and DL_CO_, expressed as absolute values and as a percent of predicted value for age, sex, and stature [15, 16]. Furthermore, GAP scores, incorporating a composite of gender, age and physiology were also calculated for each participant. Scores range from 0 to 8, whereby an increased score is indicative of a greater risk profile for early mortality [17]. Physical activity status was subjectively assessed using the General Practice Physical Activity Questionnaire [18].

### Cardiopulmonary exercise testing

Participants underwent a CPET on an electronically braked cycle ergometer (Lode Excalibur; Lode, Groningen, the Netherlands), whereby the protocol incorporated an initial warm-up at 0 W for three minutes before an incremental ramp phase increased resistance by 10 W min^−1^. Participants were instructed to maintain a self-selected cadence between 60 and 80 revolutions per minute (rpm) until volitional exhaustion, defined as a decrease in cadence < 10 rpm for 5 consecutive seconds despite verbal encouragement from research staff. Upon exhaustion, the resistance was removed, and participants returned to pedalling at 0 W for a further three minutes to cool down. This protocol has been detailed previously [14].

**Fig. 1:**
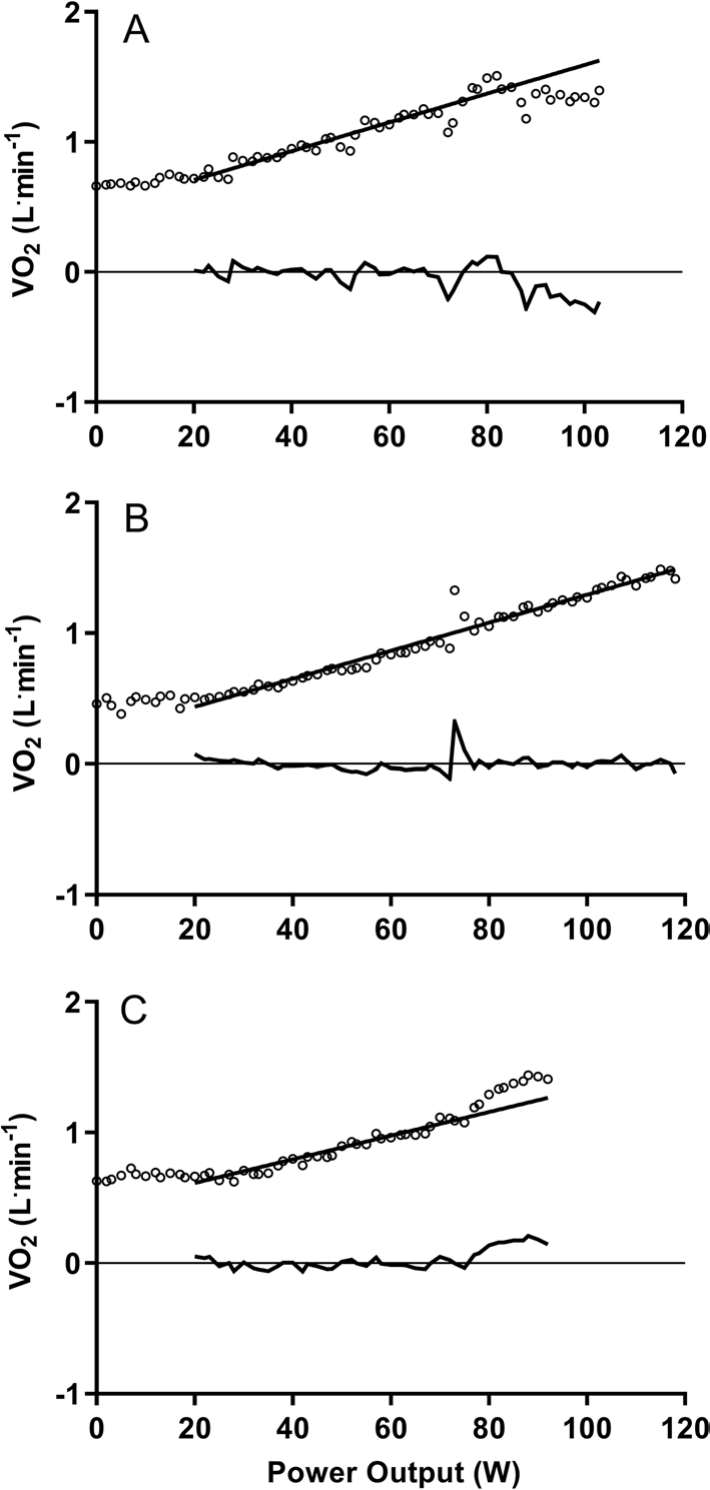
Example VO_2_ responses to increasing work-rate during cardiopulmonary exercise tests. **A**: Deceleration of VO_2_, producing a plateau (64-year old male with idiopathic pulmonary fibrosis); **B**: Linear response (70-year old male with idiopathic pulmonary fibrosis); **C**: Acceleration of VO_2_ against power (58-year old male with chronic hypersensitive pneumonitis). For all cases, the extrapolated regression line is fitted from 120 s, through to volitional exhaustion. VO_2_: oxygen uptake

Throughout the CPET, measures of pulmonary gas exchange were recorded using a metabolic cart (Medgraphics Ultima; Medical Graphics UK Ltd., Gloucester, UK), calibrated for volume and gas concentrations prior to each test. Data was measured breath-by-breath and analysed in 10 s averages, with VO_2peak_ and presence of a plateau in VO_2_ being determined using methods described previously [19]. Briefly, a linear regression was plotted over the ‘linear’ portion of the exercise test, with data from the first and last two minutes prior to exhaustion (or clinical termination) excluded. The VO_2_ from this linear portion was then extrapolated over the remainder of the test, and residuals from final 60-s isolated and examined against the extrapolated portion. A negative residual indicated a deceleration in VO_2_ against power output and was defined as a plateau when the magnitude of residuals was ≥ 5% of projected VO_2_ (Fig. 1a). Either a positive or negative residual < 5% of projected VO_2_ indicated a linear response (Fig. 1b). Finally, a positive residual ≥ 5% indicated an acceleration in VO_2_ against power output (Fig. 1c).

Normative values of Jones et al. [20], as suggested by the European Respiratory Society (ERS) [12], were utilised to present VO_2peak_ and peak work rate (WR_peak_) as a percent of predicted. Determination of the gas exchange threshold (GET) was undertaken using the V-slope method as previously described [21], and verified using ventilatory equivalents for oxygen (V_E_/VO_2_) and carbon dioxide (V_E_/VCO_2_).

Subjective ratings of perceived exertion (RPE) and dyspnoea (RPD) were recorded at baseline, throughout the CPET, and at test termination, on validated scales of 6–20 and 0–10 respectively [22].

Participants also wore a 12-lead ECG (Welch Allyn CardioPerfect; Hillrom, Chicago, USA) and pulse oximeter (Choice MMed MD300C2; ChoiceMMed, Dusseldorf, Germany), to monitor cardiac changes and peripheral capillary oxygen saturation (SpO_2_) respectively. All CPETs were supervised by an exercise physiologist and medical doctor, and the CPET was terminated if either ECG (e.g., arrhythmia) or SpO_2_ responses warranted early cessation for patient safety. In the first round of CPETs, SpO_2_ limit was set at < 88%, and extended to < 80% in the second and third CPETs as hypoxemia was shown to be well tolerated in the first CPET.

### Determination of validity

CPET was determined to be a ‘maximal’ effort (and therefore valid) if it satisfied at least one of the criteria set forward by a recently published technical standards document from the ERS [12]. With relation to the current study design (cycle ergometry with ramp protocol) and available measures (pulmonary gas exchange, work rate, retrospective spirometry and cardiac function), these criteria included a primary criterion of a plateau in VO_2_ (as previously described), or one of numerous secondary criteria, including: (1) achieving predicted VO_2peak_, using aforementioned normative equations of Jones et al. [20]; (2) achieving predicted WR_peak_, using normative equations of Jones et al. [20]; (3) achieving predicted maximal heart rate (HR_max_; calculated as 220-Age); (4) peak ventilation (V_E_) reaching, or exceeding, 85% of estimated maximal voluntary ventilation (MVV; calculated as FEV_1_ × 40); and (5) respiratory exchange ratio (RER) ≥ 1.05.

### Determination of repeatability

For participants who performed at least two valid CPETs within an either a 3-month or 6-month period (an ecologically valid time frame reflecting frequency of clinical visits), differences between CPETs were established using paired samples *t*-tests. Reproducibility was established using an existing spreadsheet [23], with calculation of (a) changes in the mean, (b) Pearson’s correlation coefficients, (c) intraclass correlation coefficients (ICC), d) absolute typical error (TE), and (e) TE expressed as a percentage of the coefficient of variation (TE_CV%_). Both TE and TE_CV%_ were calculated with 95% confidence limits and a smallest worthwhile effect size of 0.2. This approach has previously been utilised for determining repeatability of exercise based parameters in respiratory disease [24]. Furthermore, Bland–Altman analyses [25] identified the mean bias and limits of agreement (LoA) between repeated measures of VO_2_.

### Statistical analyses

Assessment of validity and repeatability have been discussed previously within the methodology and therefore, given these aforementioned approaches, no formal power calculation was undertaken. With regards to correlation coefficients, magnitudes were described as small (0.1 < 0.3), medium (0.3 < 0.5) and large (≥ 0.5) as per existing thresholds [26]. For all analyses, statistical significance was set at *p* = 0.05.

## Results

### Participant characteristics

**Fig. 2:**
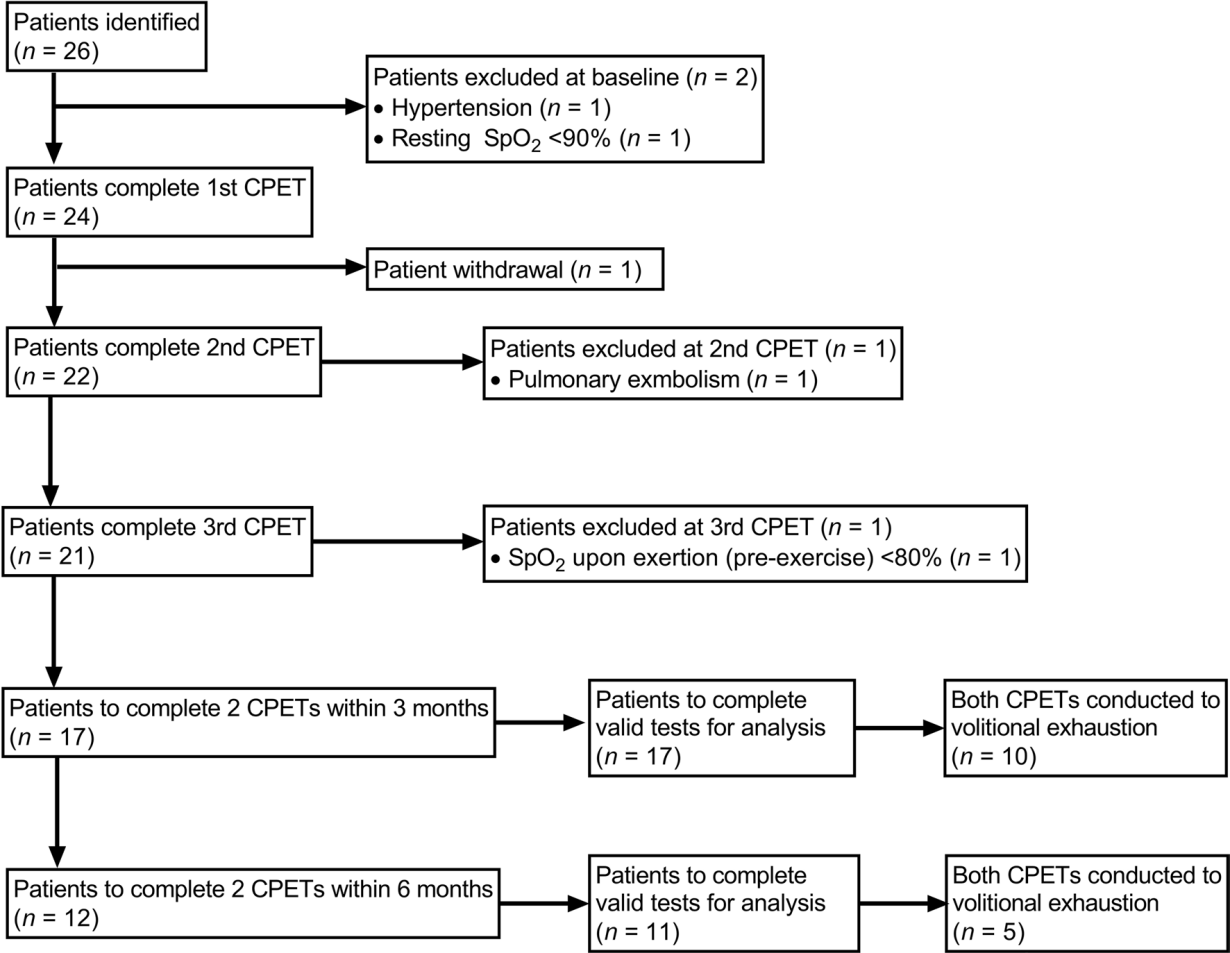
Flowchart detailing participant enrolment and successful completion of cardiopulmonary exercise tests within study. CPET: cardiopulmonary exercise test; SpO_2_: peripheral capillary oxygen saturation

Twenty-six participants were recruited, although clinical contraindications resulted in *n* = 2 being excluded from baseline CPETs, as described in Fig. 2, and described previously [14].

Therefore, *n* = 24 undertook at least one CPET. Further exclusions during the course of the study period resulted in *n* = 21 completing all three CPETs. Descriptive participant characteristics of the *n* = 26 recruited, *n* = 24 to undertake at least one CPET, and *n* = 21 to perform all three CPETs are listed in Table 1. A total of 67/78 prospective CPETs were completed during the course of this study.

### Testing timeline

For *n* = 21 participants to undertake all three CPETs, the mean duration between visits 1 and 2 was 26 ± 12 weeks (range = 12–49 weeks), and between visits 2 and 3, this was 15 ± 5 weeks (range = 10–28 weeks). Time between the first and last CPET was 41 ± 14 weeks (range = 23–65 weeks).

A total of *n* = 17 participants successfully performed at least two CPETs within a 3-month period (14 ± 2 weeks, 11–16 weeks), with this either being between the first and second CPET, or between the second and third. If participants had a 3-month gap between their first and second, as well as second and third CPETs, data between the first and second CPETs was carried forward for repeatability analyses. Within this sample of *n* = 17, a further *n* = 12 participants successfully performed two CPETs within a 6-month period (27 ± 1 weeks, 25–29 weeks).

Factors that prevented all participants completing CPETs within the prescribed 3-month (and subsequent 6-month) periods included personal availability, malfunctioning equipment, laboratory availability, participants forgetting to attend scheduled research visits, and staff availability (affected by general clinical rotas and hospital winter pressures).

### Changes in exercise and pulmonary function

Exercise based outcomes for the *n* = 21 to complete all three CPETs are listed in Table 2, whereby WR_peak_ ranged from 20 to 166 W in this group during the study, and VO_2peak_ ranged from 0.34 to 2.40 L min^−1^. However, due to the unplanned variances in individual testing timelines as mentioned above, each participant does have differing time frames between each CPET. Therefore, no formal analyses could be undertaken on these exercise-based parameters, and the data displayed in Table 2 are for descriptive purposes only, whereas formal repeatability analyses are presented further below.

**Table 2:** Changes in anthropometric and exercise responses at each study visit for *n* = 21 who completed all three study visits

Variable	CPET 1	CPET 2	CPET 3
*Anthropometric*			
Mass (kg)	82.5 ± 13.4	81.4 ± 13.3	80.4 ± 12.7
BMI (kg m^−2^)	28.1 ± 3.6	27.8 ± 3.6	27.6 ± 3.5
Body Fat (%)	35.8 ± 10.3	35.2 ± 9.8^a^	35.1 ± 8.6
Fat Mass (kg)	30.0 ± 10.6	28.4 ± 9.7^a^	28.6 ± 9.1
Fat Free Mass (kg)	52.5 ± 9.1	51.0 ± 8.4^a^	51.6 ± 8.4
*Pulmonary Function*			
FVC (%_Predicted_)	84.4 ± 17.8	82.6 ± 17.1^c^	75.7 ± 19.0^e^
DL_CO_ (%_Predicted_)	55.2 ± 12.3^b^	51.7 ± 9.8^d^	50.6 ± 12.7^d^
*Exercise*			
VO_2peak_ (L min^−1^)	1.32 ± 0.41	1.24 ± 0.50	1.15 ± 0.42
VO_2peak_ (mL kg^−1^ min^−1^)	16.2 ± 2.4	15.3 ± 5.9	14.4 ± 5.2
VO_2peak_ (mL kgFFM^−1^ min^−1^)	25.0 ± 6.5	23.1 ± 8.2^a^	21.6 ± 6.6
VO_2peak_ (%_Predicted_)	73.0 ± 21.6	68.7 ± 22.6	65.7 ± 22.9
WR_peak_ (W)	92 ± 39	89 ± 39	90 ± 39
WR_peak_ (%_Predicted_)	61.3 ± 22.0	60.5 ± 23.4	62.1 ± 22.9
GET (L min^−1^)	0.82 ± 0.20^f^	0.86 ± 0.22^f^	0.84 ± 0.15^ g^
GET (%VO_2peak_)	53.0 ± 10.2^f^	55.6 ± 10.9^f^	59.6 ± 11.6^ g^

All variables reported as mean ± standard deviation

*BMI* body mass index; *CPET* cardiopulmonary exercise test; *DL*_*CO*_ diffusion capacity for carbon monoxide; *FFM* fat free mass; *FVC* forced vital capacity; *GET* gas exchange threshold; *VO*_*2peak*_ peak oxygen uptake; *WR*_*peak*_ peak work rate

^a^*n* = 19 due to lack of body composition data, arising from of equipment malfunction

^b^*n* = 20

^c^*n* = 18

^d^*n* = 15

^e^*n* = 19, all due to missing spirometry data from patient records

^f^*n* = 18 for GET due to non-detection of threshold

^g^*n* = 16 for GET due to non-detection of threshold

Furthermore, due to the retrospective nature of obtaining pulmonary function data, a number of data points could not be retrieved from participant medical records, leading to incomplete pulmonary function data as seen in Table 1. There was also wide variability in the time difference between pulmonary function tests and CPETs. The smallest difference was zero days, whereby a participant had undertaken pulmonary function testing on the same day as a CPET. The mean difference was -32 ± 96 days (− 0.09 ± 0.26 years), indicating that pulmonary function tests, were as an average, undertaken 1 month prior to each CPET. However, the total range was from − 252 to 317 days (− 0.69 to 0.87 years). Therefore, given this disparity in timelines, and the fact that pulmonary function is not a primary outcome variable within this study, these data are only utilised as a descriptive variable in Table 1, and no further analyses are undertaken with regards to FEV_1_, FVC or DL_CO_. Further to these changes in pulmonary function, no mean change was identified in GAP score, or physical activity status.

### Validity of cardiopulmonary exercise testing

**Fig. 3:**
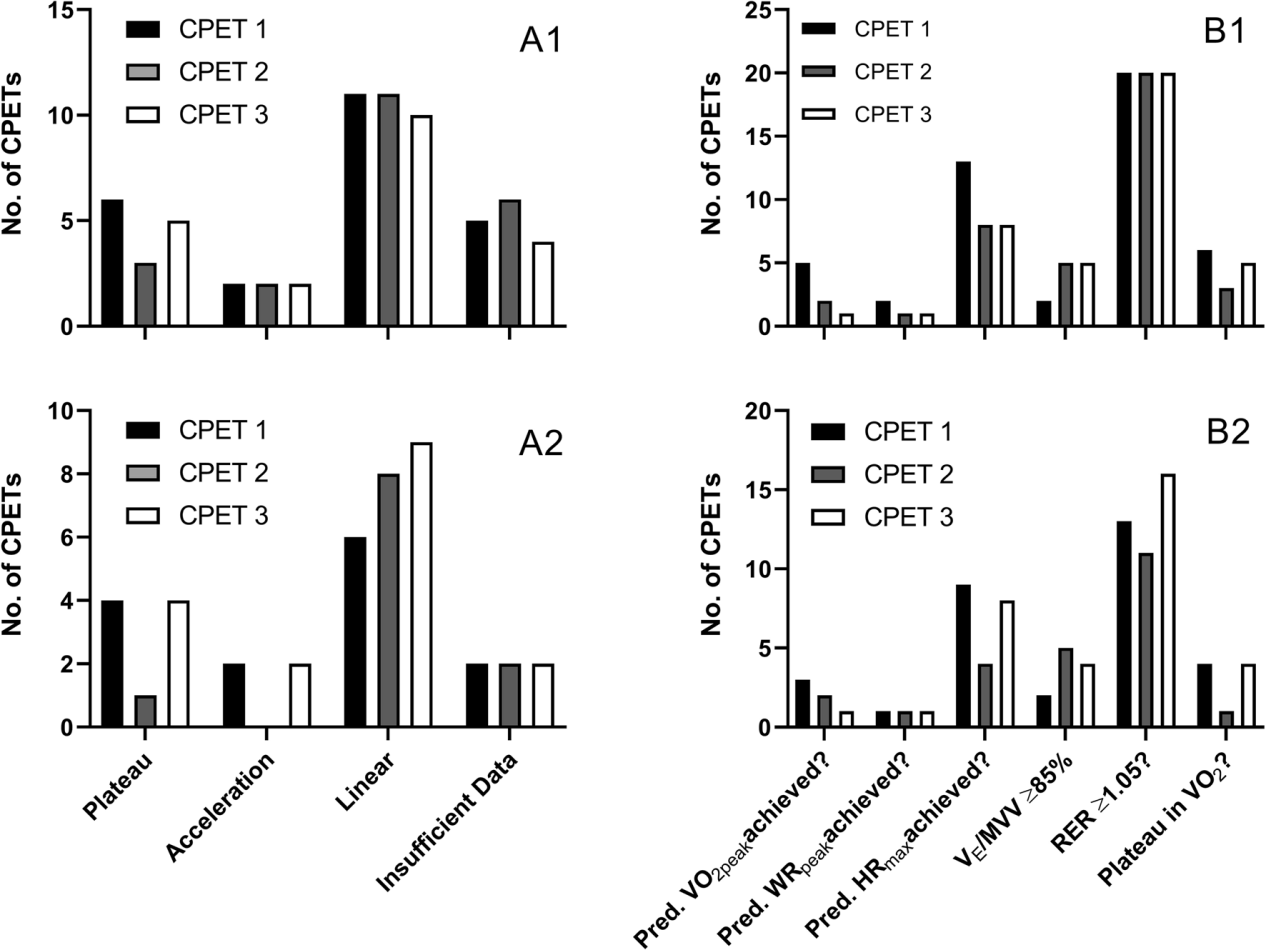
Frequency of VO_2_ profiles, and primary and secondary verification criteria, in each cardiopulmonary exercise test. 1 = All completed CPETs (CPET 1, *n* = 24; CPET 2, *n* = 22; CPET 3, *n* = 21). 2 = CPETs whereby participants reached volitional exhaustion (CPET 1, *n* = 14; CPET 2, *n* = 11; CPET 3, *n* = 17). CPET: cardiopulmonary exercise test; HR_max_: maximal heart rate; RER: respiratory exchange ratio; V_E_/MVV: minute ventilation/maximal voluntary ventilation; VO_2_: oxygen uptake; VO_2peak_: peak oxygen uptake; WR_peak_: peak work rate

The majority of CPETs undertaken were terminated due to patients successfully reaching volitional exhaustion (*n* = 42), whereas the remaining tests were terminated for clinical reasons (*n* = 24), and *n* = 1 reason was not recorded. Of the *n* = 67 CPETs completed, VO_2_ responses were linear (*n* = 32, 48%), accelerations (*n* = 6, 9%) and plateaus (*n* = 14, 21%) in nature. A total of *n* = 15 (22%) CPETs were of insufficient length to analyse VO_2_ residuals. Of the *n* = 42 CPETs terminated due to volitional exhaustion, responses were linear (*n* = 23, 55%), accelerations (*n* = 4, 10%) and plateaus (*n* = 9, 21%). A total of *n* = 6 (14%) were of insufficient length to analyse. A breakdown of these frequencies per CPET is provided in Fig. 3A1, A2.

Further to the occurrence of a number of plateaus in VO_2_, a number of secondary criteria were achieved by participants, with a full breakdown provided in Fig. 3B1, B2. Across all 67 CPETs, multiple secondary criteria were obtained: reaching predicted VO_2peak_ (*n* = 8), reaching predicted WR_peak_ (*n* = 4), reaching predicted HR_max_ (*n* = 29), reaching ≥ 85% MVV (*n* = 12) and reaching RER ≥ 1.05 (*n* = 60). At least one criteria (primary or secondary) was fulfilled for *n* = 24 CPETs, two criteria in *n* = 22, three criteria in *n* = 11, four criteria in *n* = 4 and five criteria in *n* = 2. No CPET fulfilled every primary and secondary criterion. Of the *n* = 24 CPETs terminated for clinical reasons (e.g., desaturation, cardiac contraindications), a total of *n* = 20 were verified as being maximal by reaching a number of primary or secondary criteria (1 criteria, *n* = 9; 2 criteria, *n* = 7; 3 criteria, *n* = 2; 4 criteria, *n* = 1). Of the remaining *n* = 4 that failed to present with any verification criteria, these were terminated by clinicians for desaturation (*n* = 3) and right bundle branch block (*n* = 1).

Therefore, these data indicate that 94% of performed CPETs were deemed valid. When solely considering participants who exercised to volitional exhaustion, this figure increases to 100% (42/42).

### Repeatability of cardiopulmonary exercise testing

Of the *n* = 17 participants who successfully performed two CPETs within a 3-month period, all tests were deemed to be valid, and therefore repeatability data is determined from *n* = 17. Of the *n* = 12 participants who completed two CPETs within a 3-month period, one test was deemed to be invalid, and therefore repeatability data is determined from *n* = 11.

**Fig. 4:**
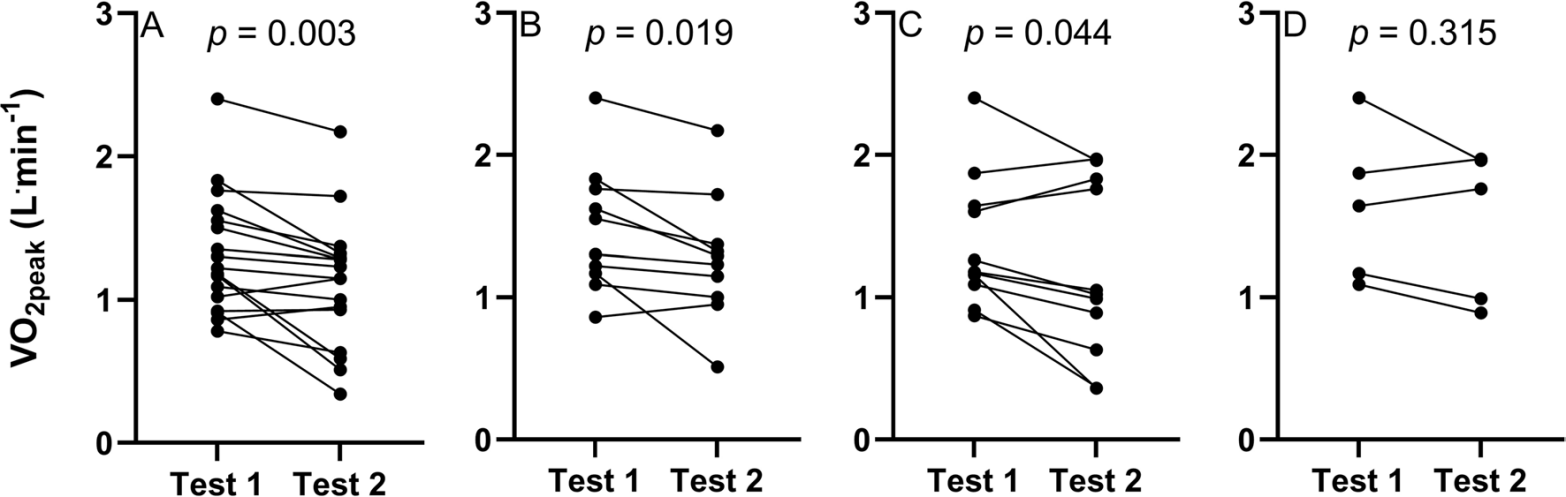
Individual changes in absolute VO_2peak_ over 3- and 6-months. **A**, **B**: three month changes; **C**, **D**: six-month changes. Data is provided for all participants (**A**, **C**) and for those only to reach volitional exhaustion (**B**, **D**). VO_2peak_: peak oxygen uptake

Statistically significant differences were seen between CPETs for all parameters of VO_2_ over a 3-month period, and most over a 6-month period, as shown in Table 3, with individual changes visualised in Fig. 4. Data in Table 3 also shows the mostly large correlation coefficients, and typical error associated with the repeatability of outcomes from each test, with this ranging from 12.7 to 25.5% over 3 months, and from 15.7 to 33.9% over 6 months, dependent on the variable being assessed.

**Table 3:** Changes in exercise responses in participants who successfully performed two valid cardiopulmonary exercise tests within a 3 month period (*n* = 17) and six month period (*n* = 11)

Variable	CPET 1	CPET 2	Mean Change	*p* Value^1^	Pearson’s *r*	*p* Value^2^	ICC	*p* Value^3^	TE (95% CL)	TE_CV%_ (95% CL)
*Three months*										
VO_2peak_ (L min^−1^)	1.32 ± 0.42	1.11 ± 0.45	− 0.21	0.003	0.85	< 0.001	0.86	< 0.001	0.17 (0.13–0.26)	25.3 (18.3–41.0)
VO_2peak_ (mL kg^−1^ min^−1^)	16.7 ± 5.1	14.0 ± 5.5	− 2.7	0.003	0.83	< 0.001	0.85	< 0.001	2.23 (1.66–3.39)	25.1 (18.2–40.7)
VO_2peak_ (mL kgFFM^−1^ min^−1^)^a^	25.9 ± 6.8	22.2 ± 8.2	− 3.8	0.004	0.83	< 0.001	0.85	< 0.001	3.19 (2.36–4.94)	25.5 (18.3–42.1)
VO_2peak_ (%_Predicted_)	79.3 ± 21.4	66.5 ± 23.1	− 12.8	0.005	0.74	0.001	0.78	< 0.001	11.41 (8.49–17.36)	24.7 (17.9–40.0)
WR_peak_ (W)	94 ± 34	87 ± 41	− 7	0.066	0.93	< 0.001	0.95	< 0.001	10.61 (7.90–16.15)	23.6 (17.1–38.1)
WR_peak_ (%_Predicted_)	67.5 ± 21.5	62.3 ± 25.6	− 5.3	0.079	0.89	< 0.001	0.93	< 0.001	8.22 (6.12–12.51)	23.7 (17.1–38.2)
GET (L^.^min^−1^)^b^	0.85 ± 0.20	0.72 ± 0.15	− 0.13	0.033	0.38	0.198	0.46	0.100	0.14 (0.10–0.23)	19.8 (13.9–34.8)
GET (%VO_2peak_)^b^	61.4 ± 10.2	60.4 ± 14.4	− 0.97	0.706	0.78	0.002	0.86	0.001	6.39 (4.59–10.56)	12.7 (8.9–21.7)
*Six months*										
VO_2peak_ (L min^−1^)	1.38 ± 0.46	1.17 ± 0.62	− 0.21	0.044	0.88	< 0.001	0.91	< 0.001	0.21 (0.15–0.38)	33.1 (22.1–65.3)
VO_2peak_ (mL kg^−1^ min^−1^)	16.9 ± 5.1	14.4 ± 6.9	− 2.5	0.050	0.85	0.001	0.89	0.001	2.66 (1.86–4.66)	32.1 (21.5–63.0)
VO_2peak_ (mL kgFFM^−1^ min^−1^)^c^	25.9 ± 6.5	20.7 ± 9.6	− 5.2	0.030	0.75	0.013	0.82	0.009	4.51 (3.10–8.24)	33.9 (22.3–70.5)
VO_2peak_ (%_Predicted_)	79.0 ± 21.3	67.1 ± 29.6	− 11.9	0.077	0.74	0.010	0.82	0.006	14.15 (9.89–24.84)	31.9 (21.3–62.5)
WR_peak_ (W)	95 ± 38	86 ± 49	− 10	0.053	0.98	< 0.001	0.97	< 0.001	10.49 (7.33–18.41)	24.6 (16.6–47.1)
WR_peak_ (%_Predicted_)	64.9 ± 19.0	58.8 ± 27.6	− 6.1	0.104	0.95	< 0.001	0.94	< 0.001	8.02 (5.60–14.07)	24.1 (16.3–46.1)
GET (L min^−1^)^d^	0.87 ± 0.19	0.85 ± 0.31	− 0.03	0.722	0.80	0.030	0.84	0.021	0.14 (0.09–0.30)	26.8 (16.5–68.6)
GET (%VO_2peak_)^d^	56.3 ± 10.6	64.5 ± 16.3	8.2	0.138	0.63	0.130	0.73	0.068	8.95 (5.77–19.71)	15.7 (9.8–37.8)

*CPET* cardiopulmonary exercise test; VO_2_peak peak oxygen uptake; *FFM* fat free mass; *WRpeak* peak work rate; *GET* gas exchange threshold; *ICC* intraclass correlation coefficient; *TE* typical error; *TECV%* TE expressed as percent of coefficient of variation; *95% CL* 95% confidence limit

^a^*n* = 17 due to lack of body composition data (equipment malfunction)

^b^*n* = 13 due to non-detection of gas exchange threshold

^c^*n* = 10 due to lack of body composition data (equipment malfunction)

^d^*n* = 7 due to non-detection of gas exchange threshold

^1^ *p* value for paired samples *t*-test

^2^ *p* value for Pearson’s correlation coefficient

^3^ *p* value for intraclass correlation coefficient

When only considering participants who reached volitional exhaustion in both analysed pairs of CPETs, statistically significant changes in VO_2peak_ are observed over a 3-month period, but not a 6 month period, although the latter is only representative of *n* = 5 participants (Table 4). Furthermore, the typical error when expressed as a coefficient of variation is lower in participants who reached volitional exhaustion (20% for absolute VO_2peak_, Table 4) than for the group which included all participants to produce a valid test (25% error for absolute VO_2peak_, Table 3).

**Table 4:** Changes in exercise responses in participants who successfully performed two valid cardiopulmonary exercise tests, whilst reaching volitional exhaustion, within a 3 month period (*n* = 10) and six month period (*n* = 5)

Variable	CPET 1	CPET 2	Mean Change	*p*-Value^1^	Pearson’s *r*	*p *value^2^	ICC	*p *value^3^	TE (95% CL)	TE_CV%_ (95% CL)
*Three months*										
VO_2peak_ (L min^−1^)	1.48 ± 0.45	1.27 ± 0.45	− 0.21	0.019	0.87	0.001	0.93	< 0.001	0.16 (0.11–0.30)	20.0 (13.4–39.5)
VO_2peak_ (mL kg^−1.^min^−1^)	17.9 ± 5.7	15.4 ± 5.9	− 2.6	0.016	0.89	< 0.001	0.94	< 0.001	1.94 (1.34–3.55)	20.6 (13.8–40.8)
VO_2peak_ (mL kgFFM^−1.^min^−1^)^a^	26.3 ± 6.3	23.6 ± 7.5	− 2.7	0.042	0.89	< 0.001	0.94	< 0.001	2.41 (1.63–4.61)	19.5 (12.8–40.7)
VO_2peak_ (%_Predicted_)	80.4 ± 17.6	69.9 ± 22.1	− 10.5	0.020	0.85	0.002	0.91	0.001	8.30 (5.71–15.16)	20.3 (13.6–40.2)
WR_peak_ (W)	105 ± 35	101 ± 43	− 5	0.238	0.97	< 0.001	0.98	< 0.001	8.50 (5.84–15.51)	19.2 (12.8–37.8)
WR_peak_ (%_Predicted_)	71.2 ± 20.2	68.5 ± 24.9	− 2.8	0.320	0.95	< 0.001	0.97	< 0.001	5.85 (4.03–10.69)	19.6 (13.1–38.7)
GET (L min^−1^)^b^	0.86 ± 0.21	0.71 ± 0.16	− 0.15	0.065	0.35	0.353	0.51	0.167	0.15 (0.10–0.29)	22.3 (14.6–47.1)
GET (%VO_2peak_)^b^	58.5 ± 11.1	59.0 ± 16.9	0.5	0.877	0.84	0.005	0.87	0.005	6.90 (4.66–13.23)	14.5 (9.6–29.6)
*Six months*										
VO_2peak_ (L^.^min^−1^)	1.63 ± 0.54	1.51 ± 0.53	− 0.12	0.315	0.90	0.035	0.95	0.007	0.17 (0.10–0.47)	10.3 (6.1–32.6)
VO_2peak_ (mL kg^−1^ min^−1^)	20.0 ± 5.9	18.7 ± 6.4	− 1.3	0.310	0.93	0.024	0.96	0.004	1.70 (1.02–4.89)	9.4 (5.5–29.5)
VO_2peak_ (mL kgFFM^−1.^min^−1^)	27.6 ± 8.7	26.0 ± 9.4	− 1.6	0.361	0.93	0.021	0.96	0.004	2.41 (1.44–6.92)	9.8 (5.7–30.7)
VO_2peak_ (%_Predicted_)	82.6 ± 19.7	78.6 ± 21.7	− 4.0	0.466	0.86	0.062	0.92	0.015	7.89 (4.73–22.67)	10.2 (6.0–32.1)
WR_peak_ (W)	113 ± 45	107 ± 59	− 6	0.384	1.00	< 0.001	0.98	0.001	10.04 (6.02–28.86)	23.9 (13.7–85.0)
WR_peak_ (%_Predicted_)	67.6 ± 26.0	65.4 ± 35.2	− 2.2	0.653	0.99	0.001	0.97	0.002	7.05 (4.22–20.26)	24.9 (14.2–89.4)
GET (L min^−1^)^c^	0.98 ± 0.14	0.94 ± 0.20	− 0.04	0.642	0.78	0.430	0.84	0.138	0.09 (0.05–0.57)	9.3 (4.8–75.3)
GET (%VO_2peak_)^c^	50.1 ± 6.0	49.1 ± 8.2	− 1.0	0.792	0.74	0.473	0.82	0.149	3.93 (2.05–24.72)	8.7 (4.4–69.0)

*CPET* cardiopulmonary exercise test; *VO*_*2peak*_ peak oxygen uptake; *FFM* fat free mass; *WR*_*peak*_ peak work rate; *GET* gas exchange threshold; *ICC* intraclass correlation coefficient; *TE* typical error; *TE*_*CV%*_ TE expressed as percent of coefficient of variation; *95% CL* 95% confidence limit

^a^*n* = 9 due to lack of body composition data (equipment malfunction)

^b^*n* = 9 due to non-detection of gas exchange threshold

^c^*n* = 3 due to non-detection of gas exchange threshold

^1^ *p* value for paired samples *t*-test

^2^ *p* value for Pearson’s correlation coefficient

^3^ *p* value for intraclass correlation coefficient

**Fig. 5:**
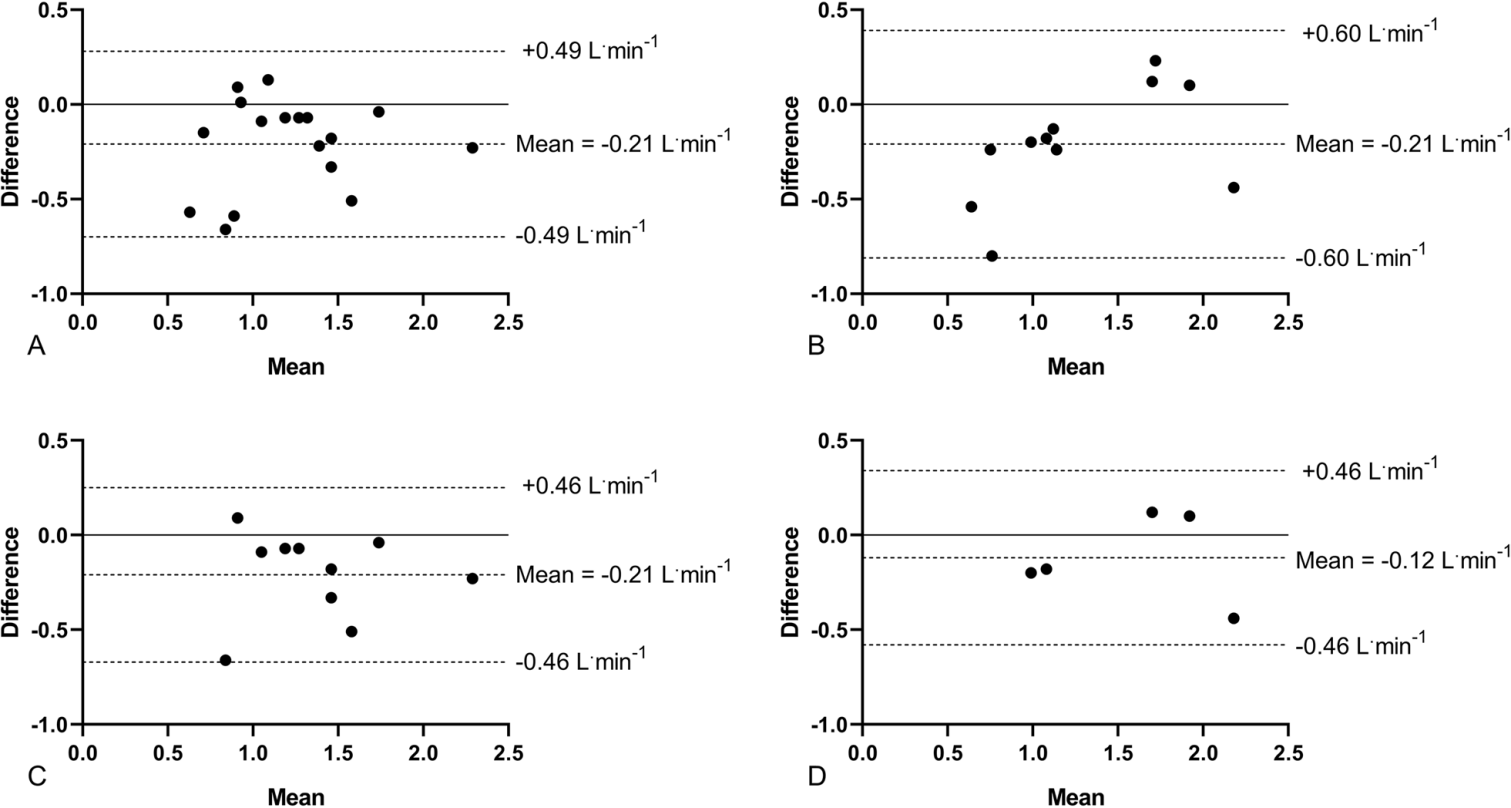
Bland Altman plots displaying mean bias and limits of agreement for absolute VO_2peak_ obtained from cardiopulmonary exercise tests. **A**: CPETs performed 3 months apart for *n* = 17 participants. **B**: CPETs performed 6 months apart for *n* = 11 participants. **C**: CPETs performed 3 months apart for *n* = 10 participants, who reached volitional exhaustion only. **D**: CPETs performed 6 months apart for *n* = 5 participants, who reached volitional exhaustion only. In each instance, difference (*y*-axis) presents data from CPET 2–CPET 1 (i.e., a value above zero indicates CPET 2 was higher than CPET 1 and therefore an increase in function has occurred). CPET: cardiopulmonary exercise test

The mean bias in absolute VO_2peak_ for the CPETs performed 3- and 6-months apart was − 0.21 L min^−1^ each, although the subsequent standard deviations and LoA differed, as shown in Fig. 5A, B. Furthermore, for participants who reached volitional exhaustion, this mean bias remained − 0.21 L min^−1^ at 3 months (Fig. 5C), with a similar limit of agreement in those to reach volitional exhaustion at 6 months (Fig. 5D).

## Discussion

This study, for the first time, has fully characterised the validity of CPET, and repeatability of associated outcomes, in a cohort of patients with ILD. This work has shown that CPET is a valid tool, whereby all participants to reach volitional exhaustion during CPET provide a valid test; and novel data has been generated surrounding the repeatability and mean bias of exercise-based outcomes over a 3- and 6-month period, with particular reference to VO_2peak_.

### Validity of cardiopulmonary exercise testing

In the first analyses of this study, focusing on the presence of a valid CPET, it was identified that 100% of participants to reach a volitional exhaustion produced a valid test, and 94% of all tests were deemed valid, even if the participant did not reach volitional exhaustion; a highly encouraging statistic. When this is combined with an expressed preference for CPET above and beyond traditional, static, pulmonary function testing [14], this highlights the ability of CPET to be integrated into respiratory services as an additional biomarker for diagnostic, prognostic, and rehabilitative reasons for an older patient group.

Traditionally, exercise studies have relied on the occurrence of a plateau in VO_2_ to determine a maximal, and therefore valid, effort [12, 27]. However, a plateau in VO_2_ does not always occur during incremental exercise, having been consistently evidenced in adults [28], children [29] and those with chronic respiratory disease [30, 31]. This is corroborated by the present study, whereby only ~ 20% of exercise tests exhibited a plateau in VO_2_. Reliance on this physiological artefact as the sole indicator of VO_2max_, and thus a maximal and valid test, is unwise as it can result in dismissal of perfectly valid and clinically useful data. Therefore, use of secondary criteria, such reaching predicted values for VO_2peak_, WR_peak_, HR_max_, maximal voluntary ventilation and the respiratory exchange ratio are also used to determine whether a maximal test has been achieved [12].

Within the present study, the majority of participants presented with an RER ≥ 1.05 and a HR_max_ exceeding their predicted value. In contrast, very few exceeded their predicted values for VO_2peak_ and WR_peak_, with this discrepancy likely due to the fact that predicted values for VO_2peak_ and WR_peak_ are generated from healthy populations and thus patient populations will simply not reach these values because of their disease status. This does not discount using VO_2peak_ and WR_peak_ values as secondary criteria, but the sole reliance upon these criteria is also not recommended, and therefore the entre CPET profile must be considered to evaluate whether a maximal effort has been reached. Further secondary criteria are available, such as changes in inspiratory capacity and blood lactate [12], and whilst these could not be assessed in the present study due to limitations of the study design, they provide a wider profile of physiological thresholds with which to determine a maximal effort.

Furthermore, it should be noted that the present study makes use of the most recent technical document for exercise testing in respiratory disease [12], whereas previous guidelines from over 15 years ago [27]—which are not wholly specific to those with chronic lung disease—utilise a slightly differing set of criteria to determine a maximal effort. The most notable differences include the presence of subjective markers of perceived effort, which are excluded from the ERS document [12]; as well as different critical threshold value for the RER. The recent ERS technical standards suggest a value of 1.05 for maximal exercise [12], whereas older from the American Thoracic Society (ATS) guidelines suggest a more conservative value of 1.15 [27]. However, within the present study, had a value of 1.15 been adopted, then only an additional four CPETs (two of which were terminated by clinicians due to desaturation) would be deemed invalid for failing to satisfy any verification criteria, thus resulting in 88% of all CPETs, and 95% of CPETs to reach volitional exhaustion, being deemed valid. Therefore, the authors believe such a change in a singular secondary verification criterion does not detract from the overall validity seen for CPET in ILD. Moreover, subjective ratings of perceived effort and dyspnoea were collated within this study, but are not formally reported as this was only undertaken to safely monitor individual changes throughout exercise, and were recorded on a 6–20 scale, and not a 0–10 scale [22] as suggested by the ATS. However, understanding the repeatability and continued clinical change in perceptual responses to exercise will be of use to both clinicians and patients alike, and should be considered for future research and incorporation into future practice.

To circumvent the reliance on secondary criteria, which are not always robust in determining whether a maximal effort has been reached [29, 32], the use of supramaximal verification testing has been proposed, whereby an additional exercise challenge is presented to participants, at a work rate that is typically in excess of that achieved during a ramp incremental test [33]. This has been shown to be effective in healthy [28] and diseased adults [31], although the feasibility and acceptability of this additional physical work in people with ILD remains unknown, and the development of an optimal protocol for performing CPET in this population should be undertaken [12].

Furthermore, whilst the majority of participants reached volitional exhaustion during their CPET, 36% of participants tests were terminated early and subsequently 4% of tests were deemed invalid due to a failure to produce sufficient maximal values. Therefore, to account for these situations whereby a clinical termination of a CPET may be required, submaximal exercise parameters should be investigated in those with ILD. Previous research in other clinical populations such as CF, heart failure and COPD, has focused on parameters of oxygen uptake efficiency (VO_2_/V_E_) and ventilatory drive (V_E_/VCO_2_) [34–37]—both of which are also important in ILD [11]. The advantage of such parameters is that these do not require a full CPET to be completed, nor volitional exhaustion to be reached, which is in contrast to parameters such as the GET. Whilst the GET is considered sub-maximal, it is normally characterised as a percentage of VO_2peak_, a process that requires participants to reach volitional exhaustion upon which to anchor the threshold, and therefore defeats the purpose of attempting to source a sub-maximal parameter. Therefore, exploration of truly sub-maximal parameters that are not dependent on maximal anchors, such as the oxygen uptake efficiency plateau [34, 38], is warranted in ILD to ascertain validity and prognostic utility.

### Repeatability of cardiopulmonary exercise testing

Within the second analysis of this study, the repeatability of multiple outcome variables associated with CPET were established, notably that of VO_2peak_. Given the importance of VO_2peak_ as a biomarker in ILD [7, 8], it is critical to be able to identify error and variation in such measures, to allow successful inferences regarding physiological decline and efficacy of therapeutic regimens to be made.

As there is a paucity of data on describing natural variation in pulmonary function [39], this study has provided valuable data in identifying variation in the equally valuable marker of VO_2peak_. This study subsequently identified that absolute VO_2peak_ presented with a typical error, when expressed as a coefficient of variation, of 20% over a 3-month period in participants to reach volitional exhaustion. Whilst this error did reduce to ~ 10% over 6 months in those to reach volitional exhaustion, this analysis was only in a sample of *n* = 5 and must therefore be treated with caution.

Previous research has predominantly focused on younger, and healthy, individuals to identify repeatability of exercise testing [40, 41], with a repeatability of CPET being established in some clinical groups, such as those cystic fibrosis [24, 42] and pulmonary arterial hypertension [43], identifying lower rates of error in VO_2peak_ than the present study. However, these studies were far shorter in length, ranging from 48-h repeatability to 6 weeks and undertaken in notably younger populations, and conditions which present with notably different pathophysiology, co-morbidities, risk factors and treatment profiles to ILD. The repeatability of CPET in ‘restrictive lung disease’ has only been evaluated once previously, observing a variation of ~ 5% in VO_2_ at peak exercise over a 28-day period [44]. However, this study from Marciniuk et al.[44] was published nearly 30 years ago, and was undertaken on only six patients, three with idiopathic pulmonary fibrosis (as per the current study), two with sarcoidosis and a single case of systemic sclerosis. Therefore, the results of this prior study should be interpreted with extreme caution, and even ignored when considering advances made in ILD management in the intervening decades. In utilising both a 3- and 6-month period of observation, we have utilised a time frame that is less burdensome than a smaller resolution (e.g., 1 week) that would require more frequent testing, whilst aligning this repeatability window with the schedule of routine clinical appointments that people with ILD have with their clinical support teams, dependent upon disease severity and trajectory [45]. As the research and clinical team were not manipulating any further treatment during this study period, all observed change would be due to disease progression and therefore the authors believe that the data presented in this study are more ecologically valid than the prior data over a 28-day time frame [44].

Further to reporting the repeatability of VO_2peak_ in this population, it must also be acknowledged that the disease trajectory of ILD is markedly different to other chronic respiratory disease, having a median survival of only 2–3 years from diagnosis [46], unlike conditions such as chronic obstructive pulmonary disease or cystic fibrosis, whereby median survival times are ~ 10 and ~ 40 years from diagnosis and birth respectively [47, 48]. This therefore calls into question whether the observed variances in VO_2peak_ are representative of ‘normal’ error that would ordinarily be observed between tests, and what is a genuine decline in physiological function. A number of participants within the present study had decrements in VO_2peak_ of > 0.5 L min^−1^ over a 3-month period, and therefore distinguishing between genuine variation and disease-driven change is of importance in ILD management, and will require corroboration of the current results to establish the true repeatability of VO_2peak_. Moreover, further studies to assess repeatability over alternative timeframes (e.g., 1 week, 1 month) are warranted; aligning with the time course of potential health changes in this population.

### Study considerations

There are a number of strengths to this study. Primarily, a robust protocol was utilised to elicit VO_2peak_ in participants though gold-standard CPET; and a thorough analysis of contemporary, internationally developed, technical standards were used to establish validity of CPET in this patient group. Moreover, choice of cycle ergometry was optimal in this group, as is not only acceptable to patients with ILD [14], but is also likely less affected by dynamic stability. As people with ILD demonstrate impaired stability (e.g. stride length, contact time) during treadmill testing [49], cycling is a preferable modality as outcomes, such as VO_2peak_, will reflect a genuine cardiopulmonary function, instead of an ability to simply balance during the test.

In addition to the choice of methodology to elicit VO_2peak_, the mathematical calculation of VO_2_ plateaus, use of multiple techniques to assess repeatability (as opposed to relying on a single correlation for example), and determination of such repeatability over multiple, ecologically valid, time frames (3- and 6-months) in an under-reported group further add to the strengths and novelty of this investigation.

In respect to limitations, it is acknowledged that the unexpected variances in individual testing timelines (due to aforementioned clinical interruptions) have reduced the number of participants available for analysis, and the subsequent lack of time-aligned pulmonary function data does not permit comparison of repeatability of VO_2peak_ and FVC. Moreover, the sole use of ramp-incremental testing could be enhanced via the use of supramaximal verification testing [33], and whilst this was not used, it does present a unique avenue for future research in this population. Despite limitations, the presented data remains novel and clinically useful for clinicians developing CPET services, and researchers utilising CPET as a tool for further investigations.

## Summary

In conclusion, this study has for the first time, fully examined, characterised, and established the validity and reproducibility of CPET within a cohort of patients with ILD. This study has utilised gold-standard testing methodologies, and existing analytical methods used previously in respiratory disease, finding CPET to be valid, and repeatable in this patient group. This data will prove useful for clinicians and researchers when using CPET as a diagnostic and prognostic tool. In particular, to investigate the utility of sub-maximal parameters in this disease group and identify repeatability of exercise-based parameters in relation to static lung function measures currently used to track disease progression is warranted.

## Data Availability

Data cannot be deposited in open access repositories for ethical reasons. Please contact the corresponding author (CAW) to discuss data access.

## Abbreviations

ATS: American Thoracic Society
BMI: Body mass index
CPET: Cardiopulmonary exercise testing
CV: Coefficient of variation
DL_CO_: Diffusion capacity of carbon monoxide
ECG: Electrocardiogram
ERS: European Respiratory Society
FEV_1_: Forced expiratory volume in one second
FFM: Fat free mass
FVC: Forced vital capacity
GAP: Gender-age-physiology score
GET: Gas exchange threshold
HR_max_: Maximal heart rate
ICC: Intraclass correlation coefficient
ILD: Interstitial lung disease
LoA: Limit of agreement
MVV: Maximal voluntary ventilation
RER: Respiratory exchange ratio
RPD: Rating of perceived dyspnoea
RPE: Rating of perceived effort
SpO_2_: Peripheral capillary oxygen saturation
TE: Typical error
TE_CV_: Typical error expressed as a percentage of the coefficient of variation
V_E_: Minute ventilation
VO_2_: Oxygen uptake
VO_2peak_: Peak oxygen uptake
WR_peak_: Peak work rate
